# Cofilin overexpression affects actin cytoskeleton organization and migration of human colon adenocarcinoma cells

**DOI:** 10.1007/s00418-012-0988-2

**Published:** 2012-07-13

**Authors:** Agnieszka Popow-Woźniak, Antonina Joanna Mazur, Hans Georg Mannherz, Maria Malicka-Błaszkiewicz, Dorota Nowak

**Affiliations:** 1 Department of Cell Pathology, Faculty of Biotechnology, University of Wrocław, Przybyszewskiego 63, 51-148 Wrocław, Poland; 2 Department of Anatomy and Molecular Embryology, Ruhr-University, 44780 Bochum, Germany

**Keywords:** Cofilin, Cell migration, ABPs, Actin cytoskeleton, Actin polymerization state

## Abstract

The dynamic reorganization of actin cytoskeleton is regulated by a large number of actin-binding proteins. Among them, the interaction of ADF/cofilin with monomeric and filamentous actin is very important, since it severs actin filaments. It also positively influences actin treadmilling. The activity of ADF/cofilin is reversibly regulated by phosphorylation and dephosphorylation at Ser-3, with the phosphorylated form (P-cofilin) being inactive. Here, we studied the effects of overexpression of cofilin and two cofilin variants in the human colon adenocarcinoma LS180 cell line. We have generated the LS180 cells expressing three different cofilin variants: WT (wild type), Ser 3 Ala (S3A) (constitutively active) or Ser 3 Asp (S3D) (constitutively inactive cofilin). The cells expressing WT cofilin were characterized by abundant cell spreading and colocalization of cofilin with the submembranous F-actin. Similar effects were observed in cells expressing S3A cofilin. In contrast, LS180 cells expressing S3D cofilin remained longitudinal in morphology and cofilin was equally distributed within the cell body. Furthermore, the migration ability of LS180 cells expressing different cofilin mutants was analyzed. In comparison to control cells, we have noticed a significant, approximately fourfold increase in the migration factor value of cells overexpressing WT type cofilin. The overexpression of S3D cofilin resulted in an almost complete inhibition of cell motility. The estimation of actin pool in the cytosol of LS180 cells expressing S3A cofilin has shown a significantly lower level of total actin in reference to control cells. The opposite effect was observed in LS180 cells overexpressing S3D cofilin. In summary, the results of our experiments indicate that phosphorylation “status” of cofilin is a factor affecting the actin cytoskeleton organization and migration abilities of colon adenocarcinoma LS180 cells.

## Introduction

Tumor cell motility, migration to distant locations and invasion crucially depend on the reorganization of the actin cytoskeleton (Sheterline et al. 1998; Webb and Horwitz 2003; Lambrechts et al. 2004). Through the regulation of actin dynamics cells can coordinate these different functions. Cellular activity and behavior are mediated by internal and external cues, which activate a number of small GTP-binding proteins of the Rho family (Hall 2005) and thus orchestrate actin filament dynamics by coordinative activation of a number of actin-binding proteins (ABPs) (Pantaloni et al. 2001). Among them, the interaction of cofilin and actin-depolymerizing factor (ADF) with monomeric and filamentous actin is of paramount importance, since they stimulate the dynamic behavior of actin filaments (Condeelis et al. 2001b). Both cofilin and ADF are able to sever existing F-actin filaments. It also positively influences actin treadmilling, however the exact mechanism of this process is described by several models and hypotheses (Carlier et al. 1997; Chen et al. 2000; Condeelis 2001; Gurniak et al. 2005; Andrianantoandro and Pollard 2006; Pavlov et al. 2007; van Rheenen et al. 2007; Kuchi et al. 2007; van Rheenen et al. 2009). The activity of cofilin is reversibly regulated by phosphorylation and dephosphorylation at Ser-3, with the phosphorylated form (P-cofilin) being inactive. It is known that LIM-kinases (LIMK 1 and LIMK 2) and TES-kinase (TESK), that inactivate cofilin, are activated by Rho-ROCK and PAK kinases (Mizuno et al. 1994; Okano et al. 1995). The corresponding phosphatase Slingshot (SSH) (Okano et al. 1995) and a member of haloacid dehalogenases, Chronofin (Gohla et al. 2005), regulate the activity of cofilin by dephosphorylation (Mizuno et al. 1994).

Furthermore, the biological pathways leading to cofilin activation are stimulated by epidermal growth factor (EGF) (Mouneimne et al. 2004, 2006). EGF is an important chemoattractant, which plays a crucial role in metastasis of mammary tumors (Wyckoff et al. 2004; Kedrin et al. 2007). Upon EGF stimulation of carcinoma cells cofilin is mobilized and activated to act locally under the cell membrane leading to a reorganization of actin cytoskeleton resulting in formation of cellular protrusions, such as lamellipodia and invadopodia. These processes together with chemotactic cues determine the direction of migration (Ghosh et al. 2004; Condeelis et al. 2005). It was previously shown that transfection of carcinoma cells with anti-cofilin siRNA (Hotulainen et al. 2005) or overexpression of a constitutively active LIM kinase domain (Zebda et al. 2000) dramatically decreases the activity status of cofilin causing inhibition of cell motility (Yamaguchi et al. 2005). Elevated levels of cofilin have been shown in *Dictyostelium discoideum,* highly invasive glioblastoma cells, and in cells derived from human breast cancer (Aizawa et al. 1996; Gunnersen et al. 2000; Wang et al. 2004; Yap et al. 2005; Yamaguchi and Condeelis 2007; Wang et al. 2007). Moreover, the level of phosphorylated, inactive cofilin was reported to be decreased in cell lines derived from T cell lymphoma (Jurkat), carcinomas from the cervix (HeLa), colon (KM12), liver (HepG2), and kidney (COS1) (Nebl et al. 1996; Subramaniam et al. 2005; Yamaguchi and Condeelis 2007).

In our previous studies (Nowak et al. 2010), parental human colon adenocarcinoma LS180 cells and their selected sublines exhibiting an increased motility and metastatic potential (Opolski et al. 1998; Nowak et al. 2002, 2005; Kieda et al. 2002) were used to investigate the expression level and subcellular localization of selected ABPs. In particular, we had analyzed the changes in expression and cellular distribution of total and phosphorylated form of cofilin.

In the present study, we used the LS180 parental cell line to study the effects of overexpression of wild-type cofilin and cofilin mutants, which differ in their biological activity. The cofilin variants allowed a more direct analysis of cofilin overexpression effects on the organization of the actin cytoskeleton and changes of the migratory ability of tested human colon adenocarcinoma cells.

## Materials and methods

### Materials

Anti-cofilin rabbit antibody recognizing synthetic peptide corresponding to human cofilin sequence (IgG fraction of antiserum) was purchased from Sigma. Anti cofilin rabbit antibody (IgG fraction of antiserum) in form of buffered aqueous solution was purchased from Sigma. It recognizes antigen of mol wt ~19 kDa. The antigen was a synthetic peptide corresponding to human cofilin sequence (amino acids 154–166). The corresponding sequence is identical in pig and rat non-muscle cofilin and differs by three amino acids from that of human and chicken muscle cofilin. Alexa Fluor^®^ 568-conjugated phalloidin and goat anti-rabbit-Alexa Fluor^®^ 488 were obtained from Molecular Probes (USA). Fetal bovine serum, trypsin, glutamine, penicillin/streptomycin, G-418 (geneticin) DMEM and OptiMEM^®^ media were purchased from Invitrogen (USA). FuGene^®^ 6 was purchased from Roche Diagnostics (Germany). DNA from calf thymus and DNase I from bovine pancreas were from Sigma. Dako^®^ cytomatic fluorescent mounting medium was obtained from Dako (Glostrup, Denmark). Matrigel™ and EGF were obtained from BD Biosciences (USA). All other chemicals were classified as analytical grade reagents.

### Cell culture

The human colon adenocarcinoma cell line LS180 was obtained from the Institute of Immunology and Experimental Therapy, Polish Academy of Sciences in Wroclaw (Poland). Originally, the LS180 cell line came from the Deutsche Krebsforschungzentrum, Heidelberg (Germany). Cells were cultivated in OptiMEM^®^ medium supplemented with 5 % fetal bovine serum. Cells were cultured in 25 cm^2^ tissue culture flasks (Sarstedt, Germany) at 37 °C in 5 % CO_2_/95 % humidified air and passaged twice a week using 0.25 % trypsin/0.05 % EDTA solution.

### EGFP-cofilin constructs and transfection procedure

The cDNAs coding for S3A, S3D and WT cofilin (described previously by Moriyama et al. 1996) in pEGFP-C2 expression vector (Clontech) were a kind gift from Dr. A.G. Weeds and S. Gonsior (Cambridge, UK). The cDNAs coding for S3A (constitutively active), S3D (constitutively inactive) and WT (wild type) cofilin were cloned in pEGFP-C2 expression vector (EGFP-linker (SGRTQIS)-cofilin) using *Bgl*II and *Eco*RI restriction sites. Its properties had been described previously (Mannherz et al. 2005). LS180 cells were cultured at 50–80 % confluence on cell culture plates (ø 35 mm) or on glass coverslips placed in 24-well plates before stable or transient transfection experiments, respectively. The transient transfection was performed by mixing 1 μg DNA with 3 μl liposome transfection reagent FuGene^®^ 6 and treating the cells according to manufacturer’s protocol. Next, to obtain clones stably overexpressing WT cofilin and the cofilin mutants, the transfected cells were cultured in medium supplemented with 1 mg/ml G-418. The clones were microscopically observed for EGFP expression and the level of EGFP-cofilin expression was analyzed in Western blotting procedures (according to Towbin et al. 1979) using anti-cofilin antibodies. For further experiments, three representative clones of the cells overexpressing each type of cofilin variant were investigated.

### Migration assay

Cell migration tests were performed using Transwell™ filters (BD Biosciences). For migration, cells stably overexpressing cofilin variants (WT, S3A and S3D cofilin) were starved for 6 h in serum free DMEM medium. The bottoms of 24-well plate were coated first with 100 μl of chemoatractant (50 % Matrigel, 20 % FBS, 30 % OptiMEM, 5 nM EGF) and next with 300 μl of serum free DMEM. Prior to assay cells (5 × 10^4^) were seeded onto rehydrated Transwell™ filters placed above the polymerized chemoatractants. After 24 h, non-migrating cells on the upper side of the filters were removed. Cells that migrated through the membrane were fixed with 4 % formaldehyde, stained with Hoechst 33342 (Molecular Probes) and counted under a fluorescence microscope. The results are presented as a relative migration factor (%), where control cells which migrated through Transwell™ filters are expressed as 100 %. The experiments were performed three times, each as an independent experiment. Each independent experiment consisted of three measurements/probes.

### Fluorescence microscopy

The cells were seeded onto sterile coverslips in six-well plates and grown for 24 h. Next, the cells were fixed with 4 % formaldehyde for 20 min at room temperature and permeabilized with 0.1 % Triton^®^ X-100 in PBS for 6 min. After fixation, coverslips were blocked for 30 min with 3 % bovine serum albumin in PBS. Then the cells were incubated for 1 h first with rabbit anti-cofilin antibody and next with goat anti-rabbit IgG conjugated with Alexa Fluor^®^ 488 (diluted 1:200 in 1 % BSA in PBS). Filamentous actin was visualized by staining the cells with Alexa Fluor^®^ 568-conjugated phalloidin for 30 min. Following incubations and washing steps coverslips were mounted in Dako^®^ cytomatic fluorescent mounting medium. The overexpression of cofilin variants and the intracellular distribution of cofilin mutants were analyzed by confocal microscopy as an expression of fusion protein (EGFP-cofilin). In each case, about 25 cells were photographed every time in three independent experiments and representative cells of every subline are presented.

### EGF stimulation

Prior to fixation with 4 %, PFA cells grown on coverslips were stimulated for 5 min with 5 nM EGF diluted in serum free OptiMEM^®^.

### Isolation of cytosolic fractions

Cells were homogenized and the cytosolic fraction was prepared as described earlier by Malicka-Błaszkiewicz and Roth (1981). The cells stably overexpressing cofilin variants, grown in tissue culture dishes were gently washed with PBS, scraped with a rubber policeman, and suspended in freshly made monomeric actin stabilizing buffer, containing 10 mM Tris–HCl, pH 7.4; 1 mM dithiothreitol; 0.1 mM ATP; 0.1 mM CaCl_2_; and 0.25 M sucrose (buffer A). Cells were centrifuged (100×*g*, 3 min, 4 °C) and homogenized with 3 volumes of freshly made buffer A with a Dounce homogenizer. Homogenates were centrifuged at 105,000×*g* for 1 h at 4° C. High-speed supernatants were used as cytosolic fraction and stored at −70 °C for further experiments.

### Western blot analysis

Protein concentration in cytosolic fractions was determined by Bradford (1976) procedure.

Proteins (30 μg) were separated by 12.5 % polyacrylamide gel electrophoresis in the presence of sodium dodecylsulfate (SDS-PAGE) according to Laemmli (1970), followed by transfer to nitrocellulose membrane, by the procedure described by Towbin et al. (1979). Rabbit anti-cofilin antibodies (Sigma) were used for EGFP-cofilin (42 kDa) and endogenous cofilin (19 kDa) identification. Biotinylated goat secondary anti-rabbit antibodies and extravidin–peroxidase (Sigma) were applied according to the manufacturer’s protocols. Immunoreactivity was visualized with 3-amino-9-ethylcarbazole (Sigma) as a peroxidase substrate.

### Actin measurements

Cytosolic fractions were used as a source of actin. Actin was determined as the inhibitor of DNase I from bovine pancreas under standard assay conditions, as described by Malicka-Błaszkiewicz and Roth (1981). The concentration of monomeric actin (G) was estimated by the DNase I inhibition directly in crude cytosol samples. Total actin (*T*) content was measured after dilution of the samples with G-actin stabilizing buffer until maximal inhibition of DNase I was reached. Filamentous actin (*F*) was calculated by subtracting the amount of *G* actin from the total actin (*F* = *T* − *G*). The state of actin polymerization was defined by the F-actin to G-actin ratio (*F*:*G*).

One unit of DNase I inhibitor (actin) is the amount, which decreases the activity of 20 ng of DNase I by 10 % under standard assay condition. The actin concentration was expressed in units of DNase I inhibitor per 1 mg of sample protein. The experiments were performed three times, each as an independent experiment. Each independent experiment consisted of three measurements/probes.

## Results

### The influence of EGF stimulation on cofilin and actin distribution

**Fig. 1:**
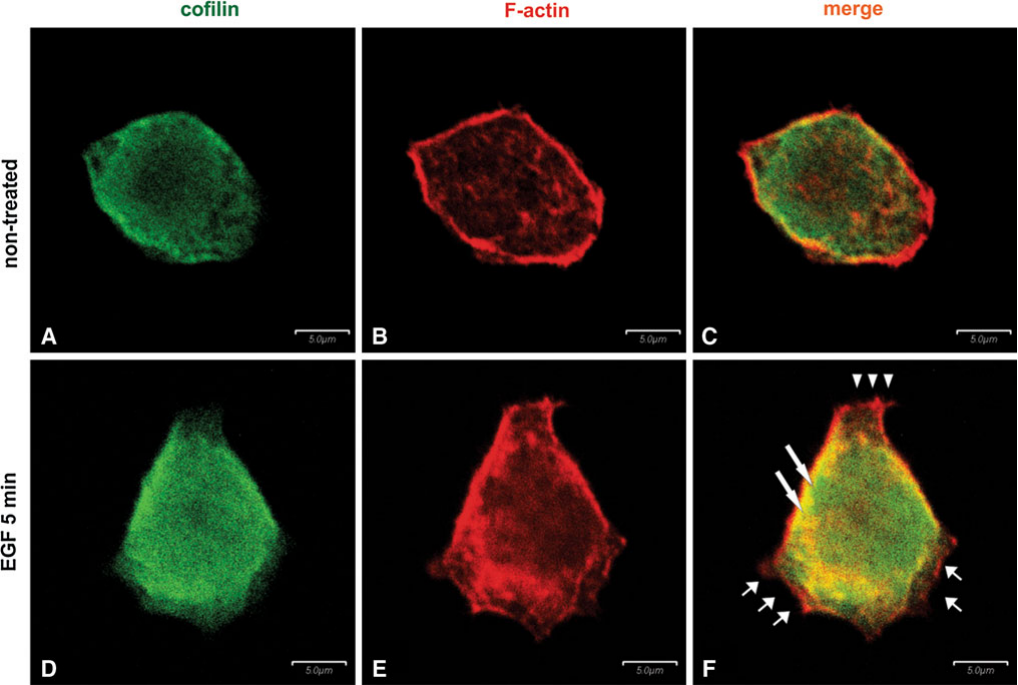
Actin cytoskeleton organization and cofilin distribution in the LS180 cells non-stimulated and stimulated with 5 nM EGF. Confocal microscopic images of LS180 cells stained with Alexa 488-labeled rabbit anti-cofilin antibody (**a**, **d**), Alexa Fluor^®^ 568-conjugated phalloidin for filamentous actin visualization (**b**, **e**), and merged images (**c**, **f**). *Long arrows* indicate the areas of presumed cofilin and F-actin colocalization. *Short arrows* indicate the areas of presumed lamellipodial membrane extensions. The *arrowheads* indicate the area devoid of submembranous cofilin localization. *Scale bar* is 5 μm

In control LS180 cells (Fig. 1a–c), cofilin was dispersed in the whole cell body (Fig. 1a). In these cells, F-actin filaments concentrated mainly in the cell periphery, most probably underneath the plasma membrane (Fig. 1b). Because EGF stimulation leads to cofilin activation in several cell lines, we tested colon adenocarcinoma cells treated with this growth factor. The LS180 cells were incubated with 5 nM EGF for 1–10 min. After 5 min of stimulation, we observed elongated cells forming probable lamellipodial extensions at their periphery rich in F-actin (Fig. 1f, short arrows). Immunostaining with anti-cofilin antibody indicated that cofilin remained mainly diffusely distributed within the cytoplasm (Fig. 1d) and furthermore that it was also localized within the presumed lamellipodial extensions, although it appeared to be accumulated in higher concentration under cell membrane, where it clearly colocalized with the high amount of F-actin (Fig. 1f, long arrows). In contrast, the opposite part of the cell was almost cofilin-negative although there was an obvious F-actin staining (Fig. 1f, arrowheads).

### The influence of cofilin overexpression on actin cytoskeleton organization

In our previous studies using LS180 parental human colon, adenocarcinoma cells and the in vivo selected variants 3LNLN and 5W cells of higher invasive potential, we have observed that the cellular cofilin pool remained as the active non-phosphorylated form (Nowak et al. 2010). Furthermore, own recent, so far unpublished data, have shown a decreased level of inactive, P-cofilin in a selected highly motile population of hepatoma Morris 5123 and human melanoma A375 cells. These interesting results prompted us to investigate the influence of the state of cofilin activity on cell migration and actin cytoskeleton organization in LS180 cells.

Therefore, we generated LS180 cells transiently and stably expressing three different cofilin variants, which were all N-terminally tagged with EGFP: WT (wild type), S3A (constitutively active) or S3D (constitutively inactive) cofilin. In S3A cofilin serine in the third position of the polypeptide chain was exchanged by non-phosphorylable residue, alanine (A), supposedly leading to a large intracellular fraction of constitutively active cofilin. In contrast, substitution of serine (S) with aspartic acid (D) in the third position of cofilin polypeptide chain introduces a negatively charged carboxyl residue that imitates the phosphorylated inactive cofilin (Moriyama et al. 1996). The observation of effects of cofilin variants overexpression on the distribution of actin filaments was performed for the LS180 cells transiently as well as stably overexpressing cofilin variants. Importantly, the effects of cofilin variants overexpression in transiently and stably transfected were the same, thus we present the results from transient transfection experiments only.

**Fig. 2:**
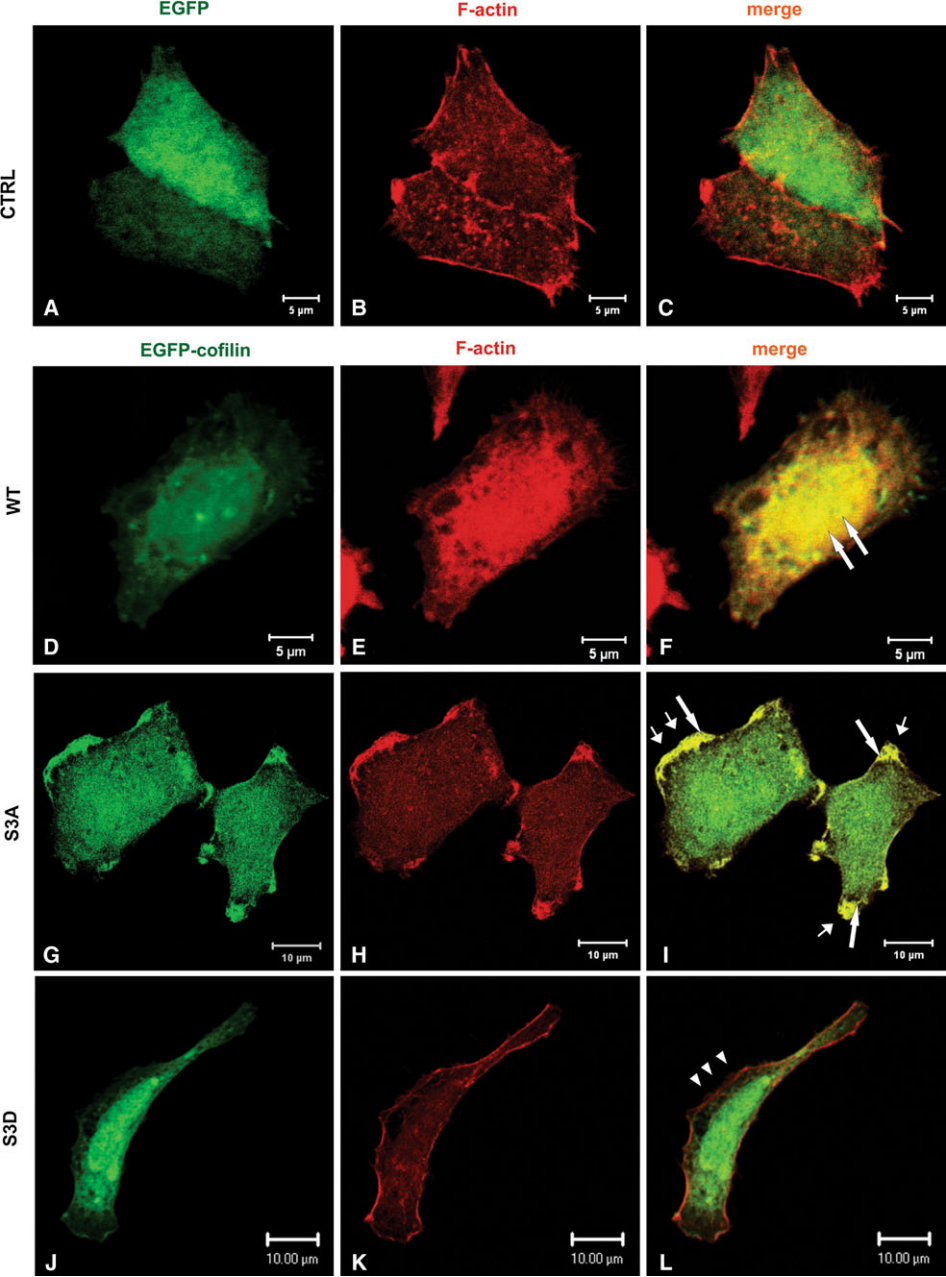
The organization of filamentous actin and cofilin distribution in LS180 colon cancer cells overexpressing different cofilin variants: WT, S3A, S3D cofilin encoded by pEGFP–C2 expression vector. The images were compared to control LS180 cells transfected with an empty vector introducing EGFP expression (EGFP). *Left panel* EGFP (**a**) or EGFP-cofilin fluorescence (**d**, **g**, **j**) (*green*). *Middle panel* filamentous actin visualized by staining with Alexa Fluor^®^ 568-conjugated phalloidin (*red*) (**b**, **e**, **h**, **k**).* Merged images* are shown in the *right panel* (**c**, **f**, **i**, **l**). *Long arrows* indicate the areas of presumed cofilin and F-actin colocalization. *Short arrows* indicate the areas of presumed lamellipodial membrane extensions. The *arrowheads* indicate the area devoid of submembranous cofilin localization (L). *Scale bar* is 5 or 10 μm

Transiently transfected LS180 cells were fixed and stained with Alexa Fluor 568-phalloidin and analyzed by scanning confocal microscopy. The results obtained for cells expressing different cofilin variants were compared to control cells (Fig. 2a–c) transfected with an empty vector introducing only EGFP expression. LS180 adenocarcinoma cells expressing WT cofilin (Fig. 2d–f) or S3A cofilin (Fig. 2g–i) were characterized by the colocalization of F-actin and cofilin (Fig. 2f, i long arrows) that was accompanied by a substantial cell spreading. Additionally cells expressing S3A cofilin formed very prominent, patchy lamellipodial protrusions (Fig. 2i, short arrows). The opposite situation was noticed for S3D cofilin-expressing cells (Fig. 2j–l). These attained a clear longitudinal morphology with F-actin distributed in submembranous area but not colocalized with cofilin that was dispersed in the whole cell body (Fig. 2l, arrowheads).

**Fig. 3:**
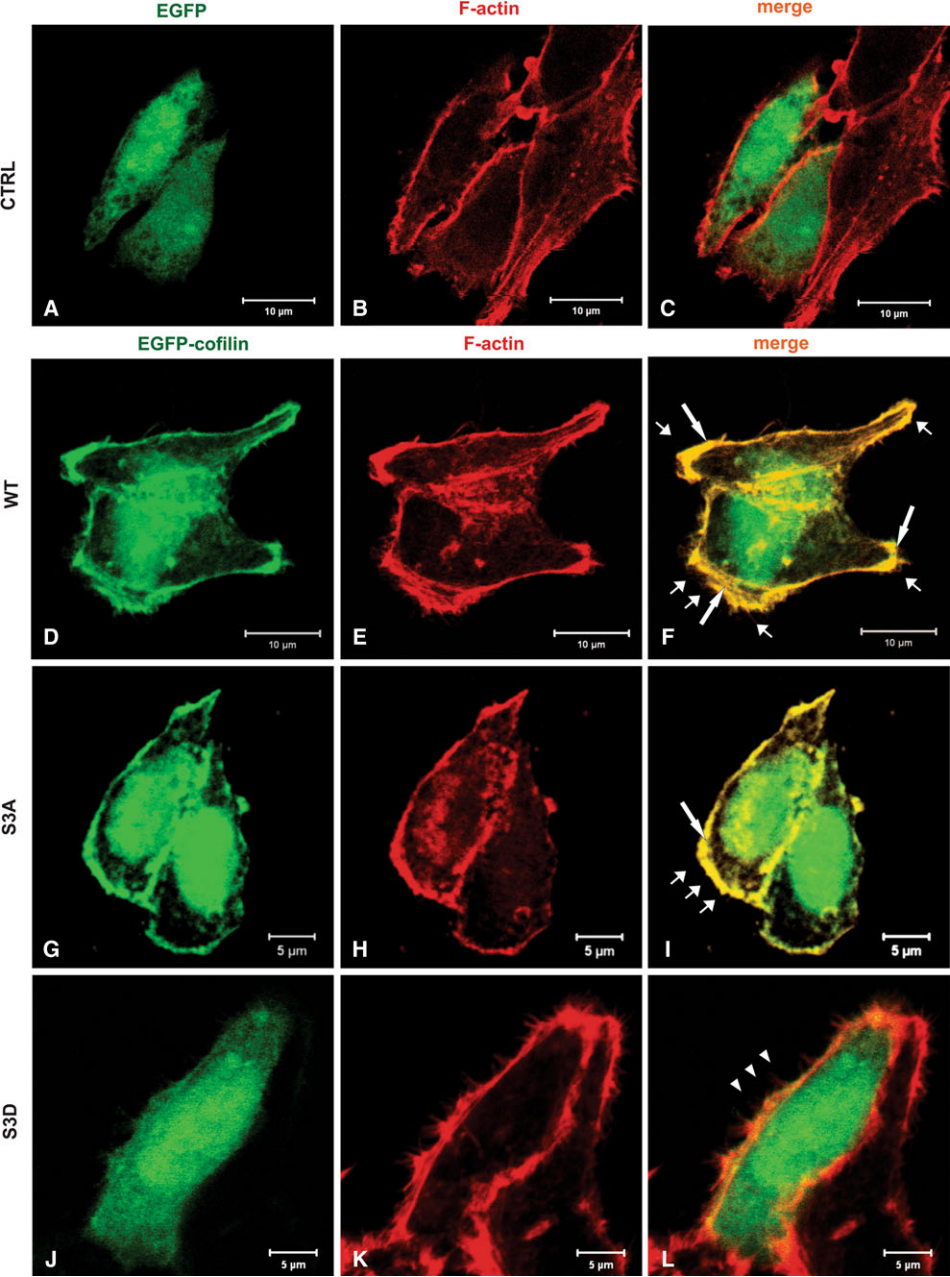
Actin cytoskeleton organization and cofilin distribution in the LS180 colon cancer cells overexpressing different cofilin variants after 5 nM EGF stimulation (5 min). Confocal images showing cells expressing the cofilin variants: WT, S3A, S3D cofilin encoded by pEGFP-C2 expression vector were compared to control LS180 cells transfected with an empty vector introducing EGFP expression (EGFP). *Left panel* EGFP (**a**) or EGFP-cofilin (**d**, **g**, **j**) fluorescence (*green*). *Middle panel* F actin (**b**, **e**, **h**, **k**) visualized by staining with Alexa Fluor^®^ 568-conjugated phalloidin (*red*). *Merged images* are shown in the *right panel* (**c**, **f**, **i**, **l**). *Long arrows* indicate the areas of presumed cofilin and F-actin colocalization. *Short arrows* indicate the areas of presumed lamellipodial membrane extensions. The *arrowheads* indicate the area devoid of submembranous cofilin (L) localization. *Scale bar* is 5 or 10 μm

Next we looked on cofilin localization and the cytoskeleton organization in LS180 cells overexpressing cofilin variants after 5 nM EGF stimulation (Fig. 3). In EGF-treated S3D cofilin-expressing cells (Fig. 3j–l), we did not observe any significant changes in cofilin and actin localization when compared with non-transfected LS180 cells (Fig. 1) or EGF non-treated transfected S3D cofilin-expressing cells (Fig. 2j–l). Cofilin in S3D expressing cells was localized within the whole cell body rather than in submembranous area and did not colocalize with F-actin (Fig. 3l, arrowheads). However, in EGF treated cells overexpressing S3D cofilin we observed high number of filipodia (Fig. 3l). In the case of WT (Fig. 3d–f) and S3A (Fig. 3g–i) cofilin-expressing cells, we noticed a visible submembranous recruitment of cofilin around the whole cell periphery strongly colocalizing with F-actin (Fig. 3f, i long arrows) in these regions of plasma membrane, which obviously possessed a lamellipodial appearance was noticed (Fig. 3f, i; short arrows).

### Cofilin activity versus cell migration ability of LS180 cells

**Fig. 4:**
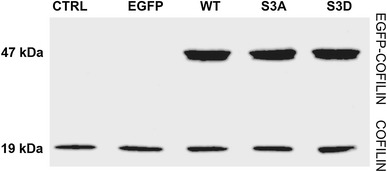
Expression of the different cofilin variants in LS180 cell. Representative immunoblot of endogenous cofilin and EGFP-cofilin variants in cytoplasmic fractions of control cells (non transfected and expressing EGFP) and cells overexpressing EGFP tagged WT, -S3A and -S3D cofilin. Equal amount of cytoplasmic fractions (30 μg of protein) obtained from the cells was separated on SDS-PAGE gels followed by immunoblotting using anti-cofilin antibodies

Next we studied the migration abilities of human colon adenocarcinoma LS180 cells stably overexpressing human cytoplasmic cofilin variants (WT cofilin, S3A cofilin, S3D cofilin) fused to enhanced green fluorescent protein (EGFP). The overexpression of cofilin was confirmed by Western blot analysis (Fig. 4). Cells expressing EGFP and untransfected cells were used as controls. Several clones expressing cofilin variants were obtained. In case of each variant, three clones expressing variants of EGFP-cofilin at high and at the same time similar level were used for further analysis.

Next we analyzed the migratory behavior of the cells stably overexpressing the cofilin variants. Further characterization indicated no differences in their proliferation rate (data not shown). Their migratory ability was quantified by a modified Transwell™ migration test (see "Materials and methods") and compared to the migration behavior of control, non-transfected LS180 cells and cells transfected with a vector introducing only EGFP expression.

**Fig. 5:**
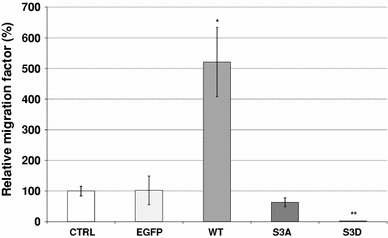
The migration ability of the LS180 colon cancer cells overexpressing the different cofilin variants: WT, S3A, S3D cofilin. Results were compared to non-transfected LS180 cells (C) and cells transfected with an empty vector introducing EGFP expression (EGFP). Cells migration ability was measured as described in "Materials and methods", using Transwell™ filters. Results expressed as the mean (±) SD are representative of at least three independent experiments. Migration in control cells is presented as 100 %. *P < 0.05, **P < 0.01 indicates value significantly different from the control cell as calculated using the Student’s *t* test

The obtained results are presented in Fig. 5. In comparison to control cells, we noticed a significant approximately fourfold increase in the relative migration factor of cells overexpressing WT cofilin. We did not observe any differences in the migration ability of the cells expressing constitutively active S3A cofilin in comparison to control and EGFP-expressing cells. However, the overexpression of S3D the constitutively inactive cofilin—resulted in an almost complete inhibition of cell motility.

### Cofilin activity affects actin pool and its polymerization state

**Fig. 6:**
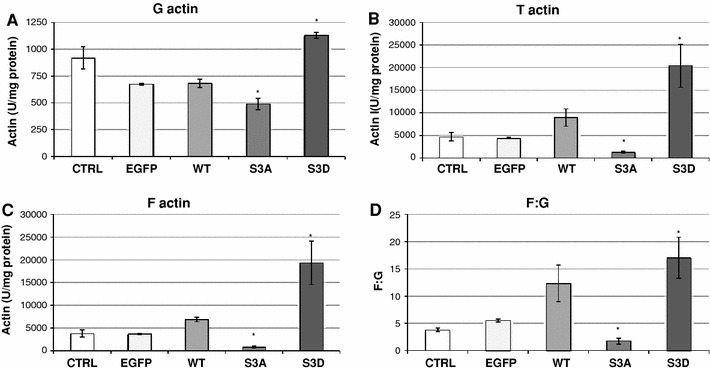
Changes in actin polymerization state in the LS180 cells expressing different cofilin variants: WT, S3A, S3D cofilin. Results were compared to non-transfected LS180 cells (C) and cells transfected with an empty vector introducing EGFP expression (EGFP). Actin was measured as an inhibitor of DNase I from bovine pancreas as described in "Materials and methods" (Malicka-Błaszkiewicz and Roth 1981). The actin concentration was expressed in arbitrary units per mg of sample protein. The different states of actin polymerization (*F*:*G*) were determined as described in the text. The *bars* represent the means (±) SD for data obtained from three independent experiments. Asterisk indicates values statistically different from those obtained for the control. A significance level was set at *P* < 0.05 in Student’s *t* test

We also focused on the determination of the state of actin pool in the cytosol of the LS180 cells stably expressing different EGFP-cofilin variants. The results of this analysis are shown in Fig. 6. In comparison to both types of control cells, the pool of monomeric (G) actin in the LS180 cells overexpressing WT cofilin did not undergo any statistically significant change and the level of filamentous (F) and total (T) actin was slightly increased. Both changes resulted in the increase of actin polymerization state in these cells. The situation was different in cells overexpressing S3A and S3D cofilin. The LS180 cells expressing S3A, constitutively active cofilin, were characterized by significantly lower levels of G, F and total T actin in reference to control cells. The opposite effects were observed in LS180 cells expressing S3D, constitutively inactive cofilin form. In these cells, we noticed an increase in monomeric G actin and in F actin pool. The changes were followed by the alterations in the level of actin polymerization state (F:G). In comparison to control cells, we determined a significantly decreased level of actin polymerization state in the cytosol of LS180 cells overexpressing S3A cofilin. In cells expressing S3D cofilin, this parameter was significantly increased.

## Discussion

The exposure of tumor cells to low concentrations of EGF stimulates cells migration (van Rheenen et al. 2007). EGF is a multifunctional growth factor, which causes activation of many cytoskeletal proteins including cofilin. Due to the localization of cofilin in the cell periphery in close apposition to the plasma membrane it increases the dynamics of actin polymerization at the cell membrane particularly in the areas, where protrusions are newly formed or already existing (van Rheenen et al. 2009).

The level of phosphorylated cofilin is low in invasive cells of the leukemic Jurkat T cell line, cervical cancer HeLa, colon KM12, liver HepG2 and kidney COS1 cells (Nebl et al. 1996; Subramaniam et al. 2005; Yamaguchi et al. 2005). It was shown that cofilin can be deactivated by LIM or TES kinase by phosphorylation of serine in position 3 of its polypeptide chain (Agnew et al. 1995; Toshima et al. 2001). Cofilin dephosphorylation should precede all the processes, for which the activity of cofilin is considered necessary. The status of cofilin phosphorylation appears, however, to differ depending on the type of cells and the kind of chemotactic factor. In neuronal cells and neutrophils, most of the cofilin pool is maintained in an inactive form until stimulated by chemotactic factors. For example, in Jurkat T-cells, SDF 1α factor causes phosphorylation of cofilin (Niwa et al. 2002). In contrast, in A431 and NIH 3T3 cells EGF stimulation causes the increase of both phosphorylated and dephosphorylated cofilin (Meberg et al. 1998). It is postulated in recent years that actin dynamics can be inhibited even in the presence of significant levels of non-phosphorylated cofilin, a presumably activated protein, and that rather the local cofilin activity at a specific compartment defines the process (Oser and Condeelis 2009). Such discrepancies make it difficult to assess the exact role of cofilin phosphorylation in biological processes. Therefore, we attempted to determine the effects of cofilin phosphorylation on the migration ability of colon adenocarcinoma cells in an in vitro model.

For this purpose, we overexpressed wild-type (WT) EGFP-cofilin and its variants: a constitutively active (S3A) and inactive (S3D) mutant form (Moriyama et al. 1996) in human colon adenocarcinoma LS180 cells. The substitutions of cofilin Ser-3 are characterized by the diverse activities of cofilin itself and its increased affinity towards actin, since S3A-cofilin binds both F-actin and G-actin. Because the alanine residue does not posses a free hydroxyl group, there is no possibility of adding a phosphate group at the third position of S3A cofilin resulting in a constitutively active state of cofilin. In contrast, substitution of serine (S) with aspartic acid (D) in the third position of cofilin polypeptide chain introduces a negatively charged carboxyl residue that causes reduction of cofilin affinity for either G- or F-actin actin (Moriyama et al. 1996). Such a tool appears to be an interesting choice for studying the impact of cofilin activity on the phenotype and properties of tumor cells. The activity of cofilin variants in mammalian cells was examined by different authors (Andrianantoandro and Pollard 2006, Lai et al. 2008). First, purified, EGFP-tagged cofilin quenched the fluorescence of pyrenylated actin filaments in a manner indistinguishable from untagged cofilin (Andrianantoandro and Pollard 2006), demonstrating actin filament binding in vitro (Lai et al. 2008). In addition, co-immunoprecipitations showed interaction of all expressed EGFP-tagged cofilin variants with actin in cell extracts, although, as expected, with less efficiency for the inactive variant (Lai et al. 2008).

The latest published data also focuses on the effects of cofilin variants overexpression in other cell types. The cofilin variants WT, S3A, and S3D were for example expressed in HUVEC cells by Fazal et al. (2009) and the effect of thrombin on actin cytoskeleton organization was studied. Similarly, the overexpression of cofilin variants was engaged by Kaji et al. (2008) to present the role of LIM-kinase mediated cofilin phosphorylation in spindle positioning in HeLa cells. Using similar approach, Shi et al. (2009) presented the role of cofilin in maintaining the morphology of dendritic spines. Oleinik et al. 2010, by analysing the cells overexpressing S3A or S3D cofilin mutants have demonstrated that FDH, a folate enzyme with suppessor like properties, inhibits cell motility via dephosphorylation of cofilin in A549 cells.

Our published data (Nowak et al. 2002, 2005, 2010) showed a correlation between higher migration ability of human colon adenocarcinoma cells, the increase in the state of actin polymerization and a low level of cofilin expression. The data presented here are in line with our previously published experimental results. A low level of cofilin expression was a factor determining the selection of our experimental model. Because the result of protein silencing is rarely absolute (Ma et al. 2009; Klemke et al. 2010; Oleinik et al. 2010), we were aware that a low level of endogenous cofilin will still remain in the cells. That’s why as an alternative we decided to overexpress different cofilin variants as a method showing the net effect of our manipulations. Our conclusions are drawn from relative experimental procedures for different cofilin variants where the level of endogenous cofilin is the same and very low.

The LS180 cancer cells overexpressing S3A cofilin produced broad lamellipodial membrane extensions rich in actin and cofilin strongly indicating that cofilin takes part in the organization of actin in these membrane structures and might thus participate in the initiation of cellular motility of these cells. Inactivation of the greater part of cellular cofilin pool by the overexpression of S3D cofilin resulted in cells unable to create lamellipodia. It also led to an elongated cell morphology. These effects could have been caused by a highly reduced affinity of the inactive cofilin towards F-actin filaments in agreement with the observed absence of colocalization of S3D cofilin with F-actin. We therefore assume that a concomitant reduction in severing activity allowed the stabilization of longer F-actin containing stress fibres leading to cell elongation, even though S3D cofilin overexpression did not influence endogenously synthesized cofilin, that could probably actively fulfill its biological functions (data not shown). Wang et al. (2006) have shown that expression of S3D cofilin in mouse MtLn3 breast cancer cells resulted in a delayed cellular response to EGF stimulation that was manifested by a greatly protracted formation of lamellipodial extensions and cell motility. Similarly, Dawe et al. (2003) observed high levels of cellular P-cofilin as a reaction to an increased level of expression of constitutively active LIM kinase, accompanied by a reduction of migratory activity and polarity of fibroblastic cells after EGF treatment, whereas restoration of cofilin activity rescued normal cell behavior and reversed these effects (Wang et al. 2006). We have also attempted to induce the overexpression of a constitutively active LIM kinase devoid of its regulatory domain in colon adenocarcinoma LS180 (data not presented). However, due to the fact that this transfection induced apoptosis we have failed to obtain stable cell clones.

Our studies showed the state of actin polymerization (*F*:*G* ratio) was elevated in cells overexpressing inactive S3D cofilin. These changes appear to be a result of the inability to attach and depolymerize actin filaments followed by a decrease in the amount of monomeric actin and increase of filamentous actin. These studies support the data obtained by Hotulainen et al. (2005) showing that cells lacking cofilin exhibit a low *G*:*F* actin ratio in reference to control cells. The *F*:*G* parameter was thus in this situation increased. In addition, Kuchi et al. (2007) confirmed that inactivation of the cellular cofilin significantly reduced the amount of monomeric actin. Here we show that high level of constitutively inactive S3D cofilin in LS180 colon cancer caused an almost complete inhibition of cell migration probably due to a significant decrease in the degree of actin polymerization state. Hotulainen et al. (2005) reported also that high cellular level of P-cofilin affects the organization of actin stress fibres and cell adhesion. These authors showed an increase in the number of atypical stress fibres and focal contacts in NIH 3T3 fibroblasts and mouse melanoma B16F cells deprived of cofilin through the use of a specific siRNA. These results were accompanied by a decrease in the pool of G actin and reduction of the migratory activity. Similar effects were observed in Neuro 2A and the N18 cells (Hotulainen et al. 2005). These studies also show that the changes in the degree of phosphorylation of a large fraction of the cytoplasmic pool of cofilin results in a decrease of the G actin pool leading to abnormally structured stress fibres.

It therefore appears that either extreme, i.e. a too high or a too low degree of actin polymerization, together with an impaired regulation of actin cycling greatly limits cell migration processes. This is in agreement with the fact that the LS180 cells overexpressing wild-type cofilin were characterized by the highest migration ability. In addition, data published by Dang et al. (2006) show that in the K1735 melanoma cells the “status” of cofilin phosphorylation depended on cell adhesion, which was mediated by cofilin binding to αvβ3 integrin receptor for vitronectin. Shortly, after cell seeding on vitronectin-coated surfaces, the level of phosphorylated cofilin was about ten times higher in cells expressing αvβ3 integrin receptor than in cells devoid of it. Furthermore, these authors also showed K1735 cells expressing high levels of wild-type cofilin exhibited a higher migration ability than control cells, what correlated with the activation of Focal Adhesion Kinase and increased expression of certain metalloproteinases.

In summary, the results of our experiments indicate that the phosphorylation “status” of cofilin is a factor affecting the morphology and migration ability of tumor cells.
